# Excited-State Chemistry of Hydroperoxymethyl Thioformate in the Troposphere

**DOI:** 10.1021/acs.jpca.5c07092

**Published:** 2026-01-26

**Authors:** David Catalán-Fenollosa, Mariana Telles do Casal, Javier Carmona-García, Alfonso Saiz-Lopez, Daniel Escudero, Daniel Roca-Sanjuán

**Affiliations:** † Institut de Ciencia Molecular, 16781 Universitat de Valencia, 46071 Valencia, Spain; ‡ Department of Chemistry, Quantum Chemistry and Physical Chemistry Division, 54517 KU Leuven, Celestijnenlaan 200F, 3001 Leuven, Belgium; § Centre for Computational Chemistry, School of Chemistry, 1980 University of Bristol, Bristol BS8 1TS, U.K.; ∥ Department of Atmospheric Chemistry and Climate, 69568 Institute of Physical Chemistry Blas Cabrera, CSIC, 28006 Madrid, Spain

## Abstract

Dimethyl sulfide (DMS; CH_3_SCH_3_) is a gas produced by phytoplankton in the ocean and emitted into the atmosphere. DMS emission is the largest source of atmospheric sulfur. Hydroperoxymethyl thioformate (HPMTF) is an oxidation product of DMS in the marine atmosphere. While the formation pathways of HPMTF are well established, the atmospheric removal processes have yet to be fully characterized. Here, we study the photochemistry of HPMTF using computational methods. Our results indicate that HPMTF photolysis is efficient (high quantum yield, ϕ = 0.67), primarily proceeding via S–C bond cleavage in the thioformate (−SCHO) group. However, it is limited by the weak absorption of UV–vis solar radiation, resulting in a long photolytic lifetime (τ ≈ 30 h). Therefore, photolysis is expected to represent a minor sink for atmospheric HPMTF.

## Graphical abstract

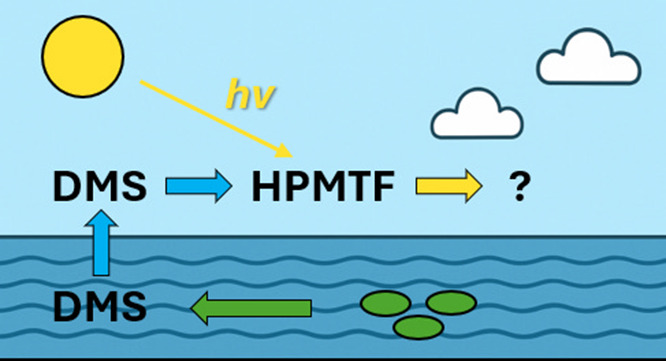

## Introduction

Sulfur chemistry exerts a substantial influence on climate.^1–5^ Dimethyl sulfide (DMS) has the largest natural emission flux among all sulfur compounds,^6^ and it is expected to increase in the future due to rising sea surface temperatures.^7^ The biological activity of phytoplankton in the oceans is the predominant source of DMS in sea-air emissions.^8,9^ In the atmosphere, DMS is oxidized yielding species such as methanesulfonic acid (MSA), sulfur dioxide (SO_2_) and sulfate (SO_4_^2–^).

A recently discovered oxidation product of DMS in the atmosphere is hydroperoxymethyl thioformate (HPMTF). Oxidation of DMS followed by hydrogen abstraction and two consecutive intramolecular hydrogen shifts (H-shifts) yields HPMTF.^10,11^ The first H-shift is the rate limiting step of the process, and it competes with reactions involving other atmospheric species such as nitric oxide (NO), hydroperoxyl radicals (HO_2_) and other peroxyl radicals (RO_2_).^10,12^ The concentrations of these species are low in the marine atmosphere, where experimental measurements have observed that HPMTF is formed.^13^

Research studies have investigated the removal of HPMTF from the atmosphere through thermal and photolytic processes. Thermal processes can be classified as homogeneous, occurring in the gas phase, or heterogeneous, occurring in condensed phases such as water and aerosols. Among homogeneous reactions, the reaction with hydroxyl radicals (OH) has been studied the most and the rate constants are consistent among each other.^10,14–16^ The estimated lifetime of HPMTF associated with the OH reaction is approximately 14 h.^17^ The reaction of HPMTF with chlorine (Cl) may be as important as the OH reaction,^18^ although no experimental rate constants have been obtained yet. Other reactions have been studied, but their contributions appear to be less significant. These reactions involve the following species: nitrate radical (NO_3_),^18^ ozone (O_3_),^17^ Criegee intermediates (e.g., CH_2_OO),^19^ and sulfur trioxide (SO_3_) catalyzed by water.^20^

The heterogeneous processes examined include: (1) reactive uptake of HPMTF to aerosol particles,^21^ (2) dry deposition,^17,22–24^ and (3) wet deposition, especially cloud uptake.^13,17,22,25^ Among these processes, cloud uptake is the dominant loss pathway for HPMTF and its estimated lifetime is less than 1–2 h.^22,25^ Although the aqueous-phase reaction mechanism remains uncertain, it is suggested that HPMTF leads to SO_4_^2–^.^22,26^

Research on the photolysis of HPMTF remains scarce. Khan et al.^23^ estimated the photolytic loss of HPMTF through compounds that share functional groups with HPMTF, herein referred to as the *chromophore approximation*. Specifically, the thioformate group (−SCHO) of HPMTF was compared with propanal (CH_3_CH_2_CHO), while the hydroperoxymethyl group (−CH_2_OOH) was compared to methyl hydroperoxide (CH_3_OOH). Therefore, the photolytic activity of HPMTF was approximated by the authors as the addition of the photolytic activity of propanal and methyl hydroperoxide. However, theoretical studies have shown that the chromophore approximation overestimates the photolysis rate constant by a factor of 3.^27^ Other works consider the photolysis as a minor loss pathway through comparison with a similar compound with a lifetime between 3.7 and 5.4 days, methyl thioformate (MTF).^14,15,17,22^

In this study, we build upon previous theoretical research that focused on the determination of the absorption cross sections. Our aim is to develop a more comprehensive picture of the photochemistry of HPMTF. We seek to (1) characterize the photochemical pathways of HPMTF through both static scans and dynamical analyses, (2) estimate its photolysis quantum yield, (3) determine the photolysis rate and the associated lifetime of HPMTF in the gas phase, and (4) evaluate the global implications of its photolysis. Additionally, we revisit and refine previous calculations, specifically the conformational analysis and its UV–vis absorption spectrum.

## Computational Details

A selection of wave function theory (WFT) methods were employed to optimize and calculate vibrational frequencies: Mo̷ller-Plesset second order perturbation theory (MP2),^28,29^ Coupled Cluster singles and doubles (CCSD),^30^ State-Average Complete Active Space Self-Consistent Field (SA-CASSCF),^31^ and Extended Multi-State Complete Active Space second-order Perturbation Theory (XMS-CASPT2).^32,33^ Ground-state geometry optimizations were performed using MP2 and CCSD, while excited-state (*S*_1_ and *T*_1_) geometry optimizations were performed using SA-CASSCF and XMS-CASPT2. XMS-CASPT2 optimizations were calculated using analytical gradients. The vertical excitation energies and oscillator strengths were obtained using the second-order approximate model of CCSD (CC2),^34^ Multi-State CASPT2 (MS-CASPT2),^35^ and XMS-CASPT2. The MS-/XMS-CASPT2 calculations considered an imaginary shift of 0.2 atomic units (a.u.)^36–38^ and an IPEA shift of 0.25 a.u.,^39^ unless otherwise specified. The basis set used for single-reference methods (MP2, CCSD and CC2) was def2-TZVP,^40^ while multiconfigurational and multireference methods (SA-CASSCF, MS-CASPT2 and XMS-CASPT2) used ANO-L-VTZP.^41,42^ MP2 and CCSD calculations were performed using Gaussian 16 A.03,^43^ CC2 calculations were performed using TURBOMOLE V7.7.1,^44^ and SA-CASSCF and MS-/XMS-CASPT2 calculations were performed with OpenMolcas v24.10.^45^

The SA-CASSCF and MS-/XMS-CASPT2 calculations were performed using two Complete Active Spaces (CAS). The first, CAS­(16,12), comprises one lone pair (*n*) orbital on each of O2, O3, S4, and O6 (see Figure 1 for atom labeling); three σ/σ* orbital pairs from the C1–S4, O2–O3, and S4–C5 bonds; and one π/π* pair on the C5–O6 bond. The second, CAS­(10,8), is a reduced subset of the CAS­(16,12) space, including lone pairs on S4 and O6, two σ/σ* orbital pairs from the O2–O3 and S4–C5 bonds, and one π/π* orbital pair on the C5–O6 bond. The molecular orbitals of the active spaces are represented in Figures S1 and S2.

**Figure 1:**
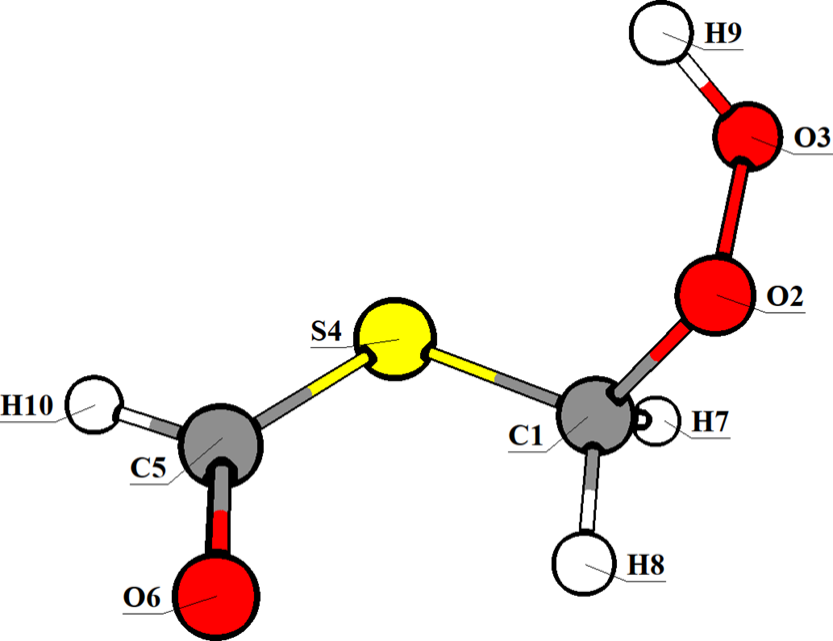
Molecular representation of the C4 conformer of HPMTF with atom numbering and labeling. The SX naming system of the conformers from ref 27 was replaced by CX in this work to avoid confusion.

Besides WFT methods, density functional theory (DFT) calculations were also performed to optimize geometries and calculate vibrational frequencies, while time-dependent DFT (TD-DFT) calculations were performed to compute vertical excitation energies and oscillator strengths. The functionals common to both methods were CAM-B3LYP,^46^ M06–2X,^47^ PBE0,^48^ and ωB97-XD,^49^ while B3LYP^50,51^ was employed only in DFT calculations. The def2-TZVP basis set was used for all calculations, except for the dihedral scans in the conformational exploration, where def2-SVP was employed. All DFT calculations were performed using Gaussian 16 A.03, while TD-DFT calculations were performed with Gaussian 16 A.03 and TURBOMOLE V7.7.1.

Potential energy surfaces (PESs) of the excited-states along selected coordinates were computed to analyze the photochemical pathways and estimate their energy barrier sizes. These pathways were obtained through geodesic interpolations between relevant molecular structures using the geodesic_interpolate software.^52^

The UV–vis absorption spectra were calculated using the Nuclear Ensemble Approach (NEA),^53^ and the calculations were performed using the tool package Multispec v1.0.^54^ For each generated spectrum, the nuclear ensemble consisted of 100 geometries obtained using a Wigner distribution at 300 K,^55^ which employed the frequencies calculated with ωB97-XD at the optimized ground-state structure. Vertical excitation energies and oscillator strengths were computed using MS-CASPT2­(16,12) and were combined with a Gaussian phenomenological broadening (fwhm of 0.2 eV) to obtain the spectrum. Gaussian 16 A.03 was used for geometry optimization and vibrational frequency calculations interfaced with Newton-X v2.2 ^56^ for NEA calculations and OpenMolcas v24.10 for the absorption energy and oscillator strength calculations.

Fewest Switches Surface Hopping (FSSH)^57^ nonadiabatic molecular dynamics (NAMD) simulations were performed with Newton-X v2.2. The molecular dynamics included four singlet states (*S*_0_, *S*_1_, *S*_2_ and *S*_3_) and were initiated at the first excited state, *S*_1_, which is the only relevant excitation in the UV–vis radiation region. An ensemble of 500 initial conditions were generated for each conformer. For each set of initial conditions, we applied an energy restriction of ±0.5 eV centered around the *S*_1_ absorption energy of the corresponding conformer optimized in its ground state. Then, the trajectories were selected stochastically based on the oscillator strength values of the *S*_1_ absorption for each structure. Overall, for the 4 conformers for which dynamics simulations were done, 2000 initial conditions were generated and finally 93 trajectories were run after applying the stochastic criterion. The mean and standard deviation values of the energy distribution for all accepted trajectories was equal to 4.60 ± 0.21 eV. Finally, the trajectories where the total energy of the system deviated more than 0.5 eV with respect to their initial value, or in one time step, were discarded. A total of 79 successful trajectories were run for 1 ps in time steps of 0.5 fs simulated using Butcher fifth order as the integration method.^58^ We assume that the system reached the ground state when the *S*_1_–*S*_0_ energy gap was equal or below to 0.3 eV. This assumption is required due to the instability of DFT in regions of high multiconfigurational character.^59^ For the FSSH simulations, the electronic structure method was TD-DFT/ωB97-XD and the software used was TURBOMOLE v. 7.7.1. The nonadiabatic couplings were calculated using the Baeck-An approximation.^60,61^

We calculated the photolysis rates using eq 1:

J=∫ϕ(λ)σ(λ)I(λ,θ)dλ 1

where *J* is the photolysis rate, ϕ is the quantum yield, σ is the absorption cross-section, *I* is the actinic flux and λ is the wavelength. The quantum yield, ϕ, was obtained from the FSSH simulations. The absorption cross-section, σ, was obtained in the absorption spectrum calculation. The actinic flux represents the intensity of the solar radiation and was estimated using the online calculator provided by the National Center for Atmospheric Research.^62^ The default values for the parameters (θ) were: solar zenith angle (0°), overhead ozone column (300 du), surface albedo (0.1), ground elevation (0 km), and measurement altitude (0 km). Precise consideration of these parameters using atmospheric models falls outside the scope of this work.

## Results and Discussion

The work is structured into two sections: ground- and excited-state electronic structure calculations. We revisit calculations presented in a previous work,^27^ and we introduce novel aspects that were not considered previously. The ground state section focuses on the conformational analysis of HPMTF, while in the excited state section we discuss the absorption spectrum and photochemistry of HPMTF and evaluate the implications of the photolysis in the marine atmosphere.

### Ground-State Conformational Analysis

Ground-state electronic structure calculations performed in our previous work included the following methods: HF, MP2, CCSD and B3LYP.^27^ The basis set across all calculations was the Pople basis set, 6–311++G­(d,p).

In the current study, we employed the same WFT methods, and we assessed a selection of DFT functionals to evaluate their potential improvements in the system description. Besides B3LYP, we here assessed the performance of CAM-B3LYP, M06–2X, PBE0 and ωB97-XD. The basis sets utilized in all calculations were the Karlsruhe basis sets, def2-TZVP. The latter basis sets were chosen in view of their enhanced quantitative performance for energies and optimized geometries, especially in combination with the DFT calculations.^40^

For ground-state optimized geometries, in the absence of experimental structural parameters for HPMTF, we compared our electronic structure method calculations to the structure obtained in ref 19 which used a higher level of theory (DF-CCSD­(T)-F12b/jun-cc-pVDZ). Optimized structural parameters and vibrational mode frequencies were computed on one of the most stable conformers of HPMTF (conformer C3). Cartesian RMSD values were calculated for the different methods and compared against DF-CCSD­(T)-F12b/jun-cc-pVDZ (see Table S1). The RMSD values for M06–2X/def2-TZVP (0.2872 Å), B3LYP/def2-TZVP (0.2771 Å) and PBE0/def2-TZVP (0.2724 Å) exhibited poor performance relative to CAM-B3LYP/def2-TZVP (0.2185 Å), ωB97-XD/def2-TZVP (0.2092 Å), MP2/def2-TZVP (0.2115 Å) and CCSD/def2-TZVP (0.1936 Å). By comparing the exchange correlation functionals performance, it can be observed that the long-range correlation is relevant to the description of HPMTF. We selected ωB97-XD/def2-TZVP for subsequent ground-state electronic structure calculations due to its accurate description of the system and low computational cost.

In ref 27, the more stable conformers of HPMTF were obtained with CREST v3.0.1 (structures S1 to S10).^63^ Here, we assess the reliability of CREST in predicting HPMTF conformers by comparing its results with those from a manual conformational exploration.

The manual conformational exploration involved sampling the torsions of the four single bonds C1–O2, O2–O3, C1–S4, and S4–C5 (Figure 1) using the ωB97-XD/def2-SVP level of theory. The resulting minima were reoptimized at the ωB97-XD/def2-TZVP level. A total of 22 conformers were found. Ten of these conformers were also identified by CREST and are shown in red in Figure 2.

**Figure 2:**
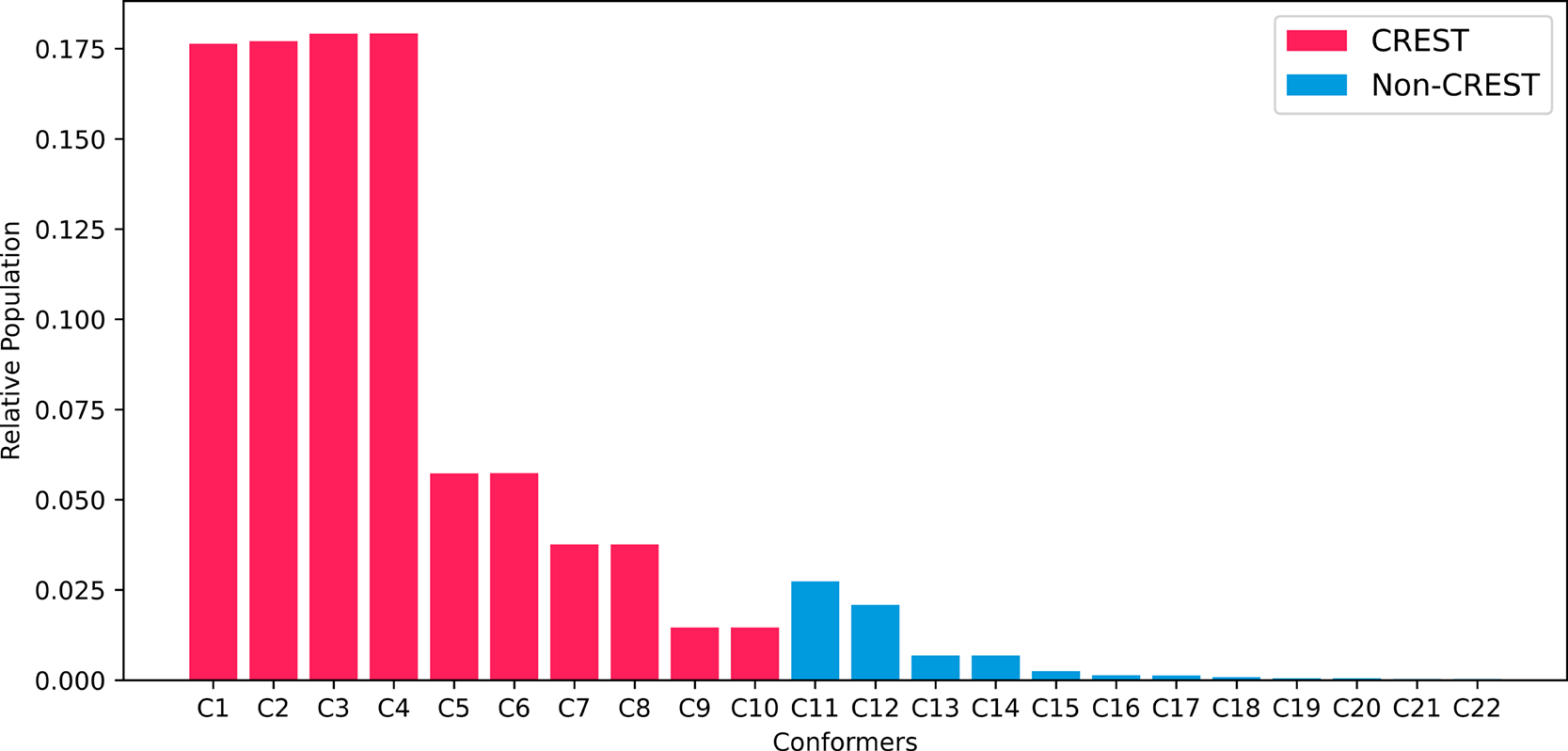
Boltzmann populations for all conformers of HPMTF at 298 K at the ωB97-XD/def2-TZVP level. Conformers with red columns were obtained through CREST and manual sampling, while blue column conformers were obtained only through manual sampling.

The Boltzmann populations of all 22 conformers were calculated using Boltzmann’s distribution formula (eq 2):

$$p_{i}=\frac{\exp\left(-\frac{\epsilon_{i}}{k_{B}T}\right)}{\sum_{j=1}^{M}\exp\left(-\frac{\epsilon_{j}}{k_{B}T}\right)}$$ 2

where *p*_*i*_ and ϵ_*i*_ are the Boltzmann population and energy of conformer *i*, respectively, *k*_B_ is the Boltzmann constant, *T* is temperature, and *M* spans over all the possible conformers. The Boltzmann populations are shown in Figure 2.

From the 22 conformers, CREST captured the eight most stable conformers and incorrectly predicted that conformers C9 and C10 were more stable than conformers C11 and C12. The error originates from the method used by CREST, GFN1-xTB, which does not accurately describe all conformers when compared with higher-level approaches such as ωB97-XD/def2-TZVP. The CREST sampling accounts for over 93% of the Boltzmann population of the HPMTF conformers at the ωB97-XD/def2-TZVP level of theory. Therefore, we considered the performance of CREST rather satisfactory. All the subsequent calculations included the ten conformers predicted by CREST only. However, for computational ease, the most expensive analyses (excited-state geometry optimizations and NAMD simulations) were restricted to the first four conformers, which account for over 71% of the Boltzmann population.

We observed that the majority of the conformers were related through pairs of pseudoenantiomers (e.g., C1/C2, C3/C4, C5/C6, C7/C8 and C9/C10). Figure 3 shows two representative pairs of conformers, C1/C2 and C3/C4. Of all conformers, only C1 and C2 exhibit hydrogen bonding (*d*_H_ ≈ 1.85 Å). All ten CREST conformers are represented in Figures S3 and S4.

**Figure 3:**
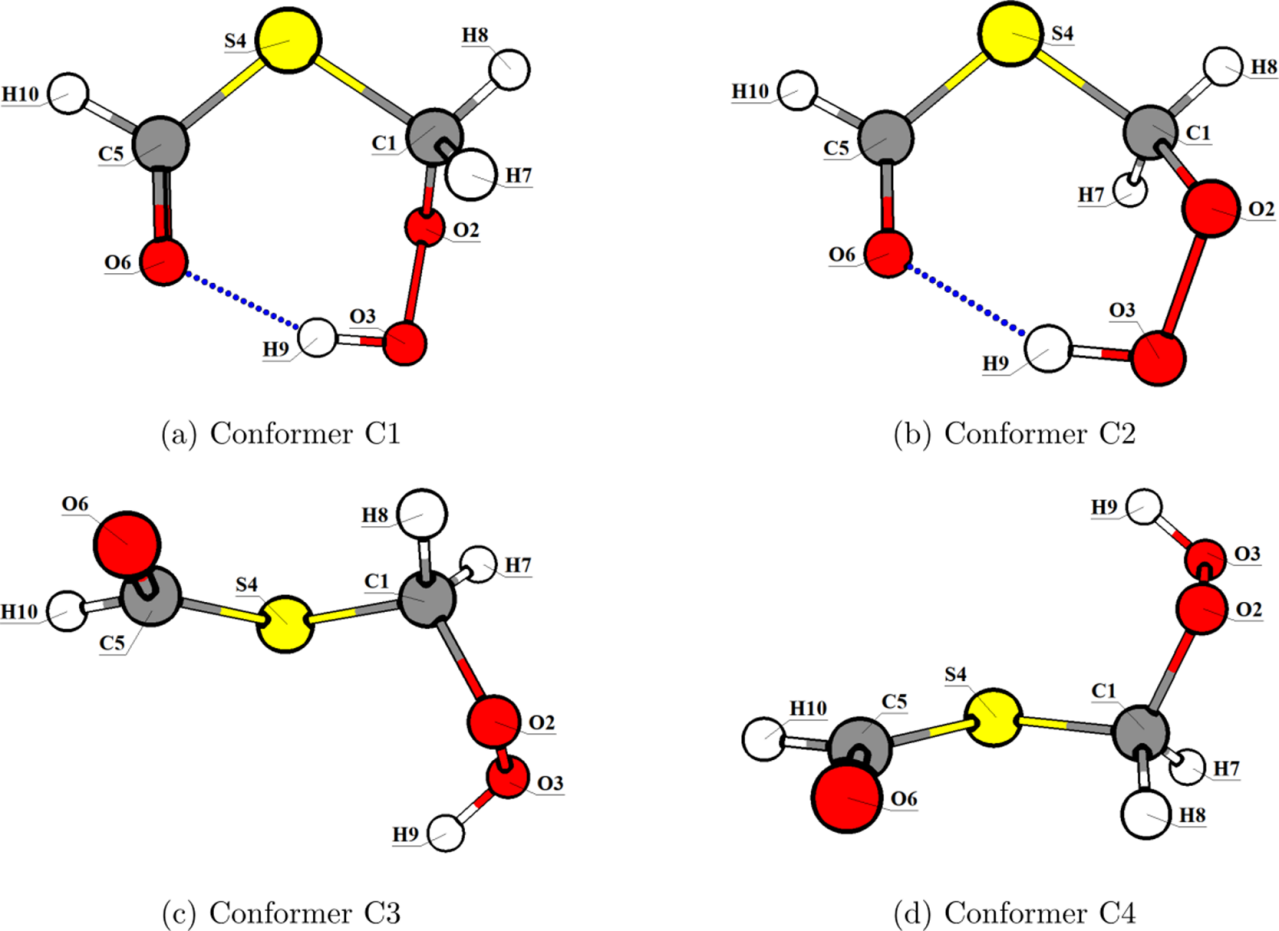
Molecular representations of the *S*_0_ minima at ωB97-XD/def2-TZVP level for conformers (a) C1, (b) C2, (c) C3, and (d) C4.

### Selection of the Methodology for Excited-State Energy Determinations

Our previous work was limited to calculate the absorption cross sections excitation energies of HPMTF, in which only SA-CASSCF and MS-CASPT2 methods were considered.^27^

In this study, in order to understand the photochemical properties of HPMTF, we need to obtain the excited-state minima, characterize the conical intersections (CIs), and simulate its excited-state dynamics. These calculations are more computationally demanding, thus we need to assess the performance of less expensive electronic structure methods.

First, we calculated the MS-CASPT2 and XMS-CASPT2 vertical excitation energies, with and without IPEA correction, for all ten conformers. The *S*_1_ absorption energies derived from these calculations are presented in Figure 4. The use of the IPEA shift remains a topic of debate.^64^ Regarding MS- and XMS- variants of CASPT2, it is established that MS-CASPT2 performs accurately in regions where the energies of the electronic states are well separated, such as the Franck–Condon (FC) region, while the XMS-CASPT2 correctly describes the avoided crossings and conical intersections where the electronic states are close to degeneracy.^65^ The purpose of doing the MS, XMS and IPEA comparisons is to quantify how large are the deviations between them and in which cases are larger. Note that agreement between them ensures highly accurate descriptions.

**Figure 4:**
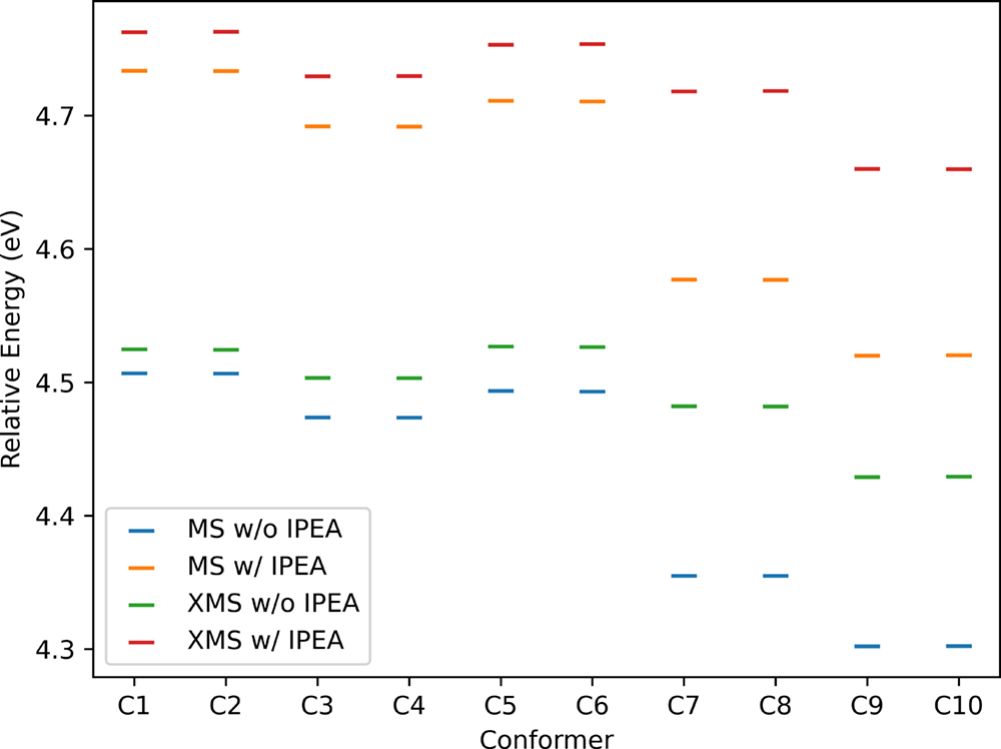
CASPT2­(16,12) *S*_1_ vertical excitation energies for all CREST conformers.

The inclusion of the IPEA shift systematically increases the *S*_1_ excitation energies by ca. 0.2 eV with respect to the calculations without IPEA correction. By comparing the MS- and XMS-CASPT2 calculations, we observe similar values for the conformers C1 to C6 (with maximum differences around 0.03 eV), while the conformers C7 to C10 had significantly different *S*_1_ excitation energies (with maximum differences around 0.15 eV). This discrepancy probably arises from the intrinsic state-averaging procedure of the XMS-CASPT2 method. Since further study of the photochemistry of HPMTF is limited to the conformers C1 to C4, we assume that XMS-CASPT2 provides accurate absorption energy values in the FC regions, and thus it is adequate for calculating potential energy curves.

The lack of experimental data prevents us from drawing further conclusions about whether the use of the IPEA correction is preferred or not. However, we expect that the behavior should be analogous to other compounds where the character of the electronic excitation is equivalent. The *S*_0_ to *S*_1_ transition corresponds to an excitation from the lone pair orbital of the atom O6 to the π* orbital of the bond C5–O6, hereafter denoted as (*n*_O_, π*). This (*n*_O_, π*) transition is characteristic to carbonyl groups and has been studied in other systems such as acrolein and acetone, where the inclusion of the IPEA correction yielded a better agreement with experimental data.^66,67^ Therefore, all our following calculations include IPEA corrections.

Second, we compare the performance of several exchange correlation functionals and of CC2 against the IPEA-corrected MS-CASPT2 vertical energies. Figure 5 represents the relative energy differences for the calculated *S*_1_ vertical excitation energies for the different methods against MS-CASPT2. All functionals underestimated the excitation energies with errors smaller than 0.1 eV, which was well within the expected error of 0.2–0.3 eV.^68^ CC2 systematically predicts larger vertical excitation energies than MS-CASPT2 by up to ca. 0.2 eV, which is consistent with the 0.2–0.3 eV error observed in benchmarks.^67,69^ Based on the reasonable performance of all methods, we selected the CC2 method and TD-DFT/ωB97-XD for further calculations.

**Figure 5:**
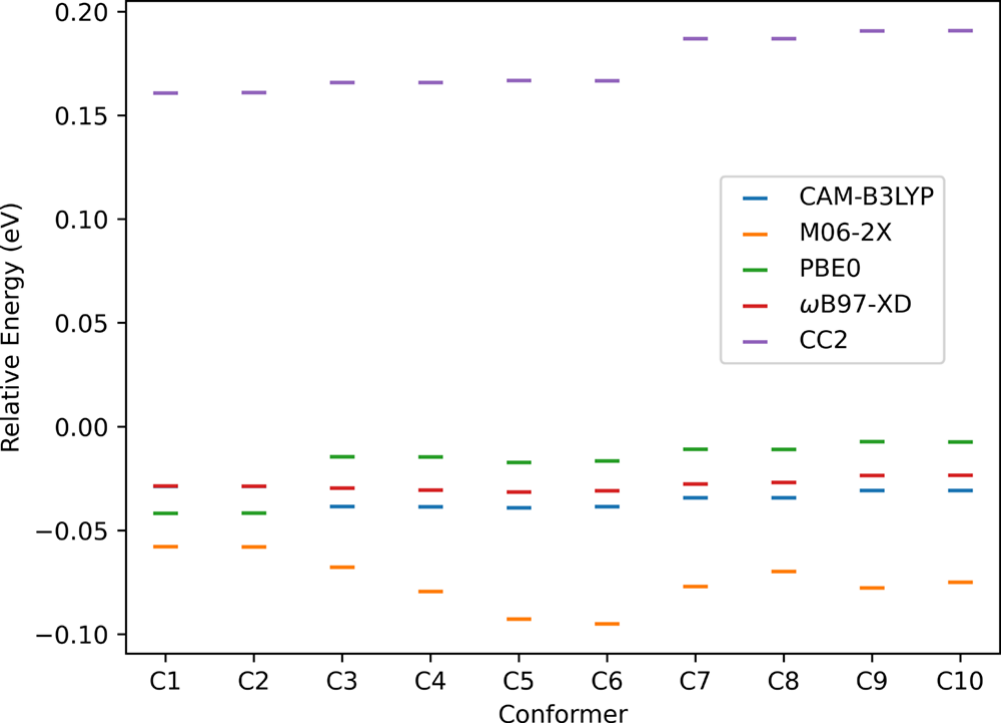
Relative energy differences for the calculated *S*_1_ vertical excitation energies for the different methods against MS-CASPT2­(16,12) for conformers C1 to C10. Exact excitation energy values are given in Table S2.

### UV–Vis Absorption Spectrum

The gas-phase UV–vis absorption spectrum was recomputed as compared to our previous work due to the change in ground-state calculations from B3LYP to ωB97-XD,^27^ which affects the UV–vis spectrum of HPMTF through two indirect sources. First, it renders different optimized structural parameters and vibrational modes, which are the basis of the NEA simulations. Second, it affects the Boltzmann populations, which determine the relative contribution of each conformer to the total spectrum.

The gas-phase UV–vis spectrum was obtained through a weighted average of the computed spectrum of each conformer. One conformer from each pseudoenantiomer pair (C1, C3, C5, C7, and C9) was included, and its weight was set equal to the sum of both Boltzmann populations. The averaged UV–vis absorption spectrum was calculated using MS-CASPT2 with the IPEA correction, and it is shown in Figure 6 together with the spectra obtained at the MS-CASPT2//B3LYP level^27^ and the chromophore approximation.^23^ The individual UV–vis spectra for each of the conformers contributing to the weighted spectrum are shown in Figure S5. We observed that conformers C5, C7, and C9 exhibit spectral shapes similar to the chromophore approximation, which is constructed as the sum of methyl peroxide and propanal UV–vis spectra, particularly resembling the propanal contribution.^27^ However, conformer C1 is drastically different due to the intramolecular H-bond formed, which does not occur in propanal or the rest of conformers. This effect is one of the fundamental reasons why the chromophore approximation is not accurate for HPMTF.

**Figure 6:**
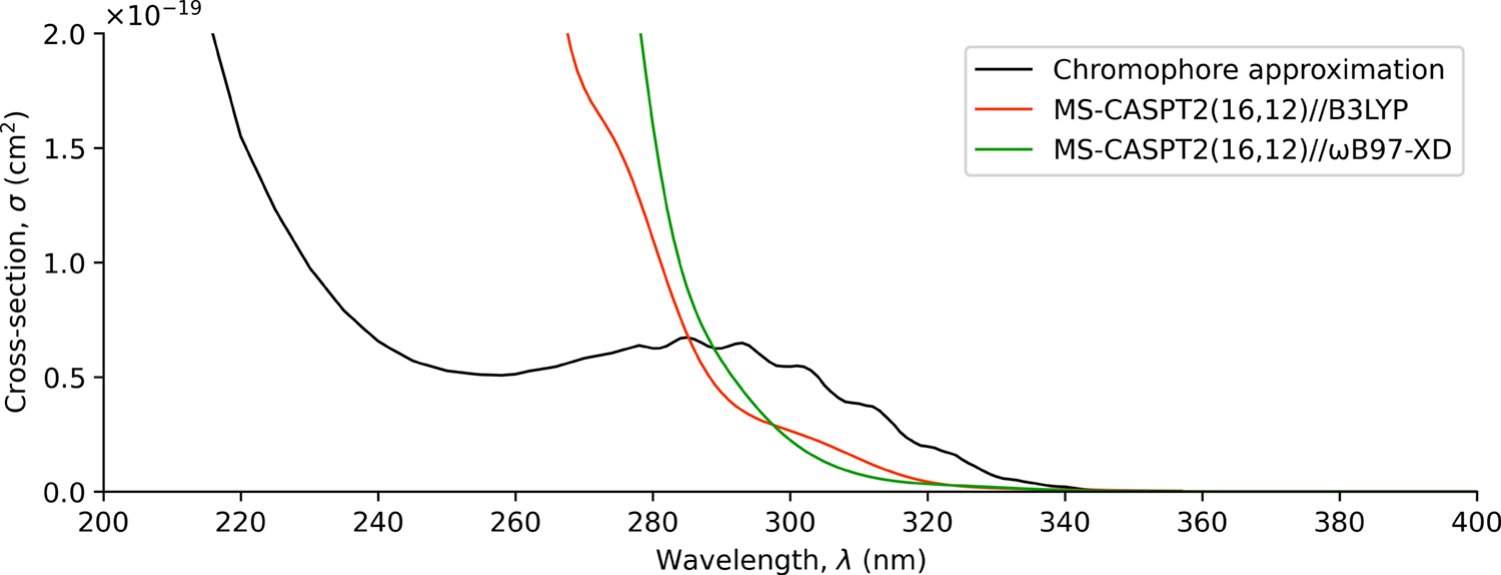
Gas-phase UV–vis absorption spectra of HPMTF. The black curve corresponds to the chromophore approximation.^23^ The red and green curves correspond to calculated spectra with NEA using B3LYP^27^ and ωB97-XD (this work), respectively.

Regarding the comparison of the spectra obtained at the MS-CASPT2//ωB97-XD and MS-CASPT2//B3LYP levels, it can be seen that overall the description is rather similar. Although the ωB97-XD spectrum shows stronger absorption than the B3LYP spectrum in the UV region below 300 nm (see Figure S6), it exhibits weaker absorption in the UV–vis region above 300 nm, where solar radiation is more intense. Upper limit estimates were calculated using eq 1 for all three spectra assuming photolysis yield equal to unity. The values and their associated lifetimes are given in Table 1. As can be seen, the differences in methodology slightly change the photolysis rates and lifetimes, though much less than more drastic strategies, such as the chromophore approximation.

**Table 1:** Photolysis Rates and Lifetimes for HPMTF Using Absorption Spectra Obtained with the Chromophore Approximation, and the NEA with MS-CASPT2 and Two Different Methods to Generate the Representative Ensemble of Geometries (B3LYP and ωB97-XD)

method	photolysis rate (s^–1^)	lifetime (h)
chromophore approximation	6.14 × 10^–5^	4.52
B3LYP	1.93 × 10^–5^	14.37
ωB97-XD	1.41 × 10^–5^	19.76

### Active Space Selection

The SA-CASSCF and MS-CASPT2 calculations performed in our previous work and in the current study, until this point, utilized the CAS­(16,12) space, herein referred to as the *extended space*.^27^ The extended space was designed to accurately describe the HPMTF. Thus, it has a large active space enabling the accurate description of higher-lying excited states, i.e., up to eight singlet excited states were calculated. This work, however, focused on the photochemistry of HPMTF and required more computationally demanding calculations. Therefore, we decreased the size of the active space for subsequent SA-CASSCF and MS-/XMS-CASPT2 calculations down to CAS­(10,8), referred to as the *reduced space*.

Specifically, since the predominant state for light absorption in the UV–vis region is the *S*_1_ state, we limited the active space to the relevant orbitals in the *S*_1_ state and other electronic states close in energy such as *S*_2_, *T*_1_ and *T*_2_, which might be relevant outside the FC region (e.g., conical intersections). The orbitals involved in the *S*_0_ to *S*_1_ transition were *n*(O6) and π*­(C5–O6) (abbreviated to *n*_O_ and π*, respectively), while the transition to the *S*_2_ state involved the latter orbitals and *n*(S4) (abbreviated to *n*_S_). The two triplet states shared similar nature to the excited singlet states. Finally, we included the sigma orbitals corresponding to the bonds O2–O3 and S4–C5 to study the bond dissociations proposed by Khan et al.^23^ The reduced active space (10,8), hereafter referred as *reduced space*, included these orbitals and the sigma orbitals corresponding to the bonds O2–O3 and S4–C5 to study the bond dissociations proposed by Khan et al.^23^ For the energetic determinations beyond the FC region, we limited the number of electronic states to four states per multiplicity (4 singlets and 4 triplets).

We computed the vertical energies for conformers C1 to C4 with the reduced space and compared them against the extended space, given in Table 2. The vertical excitation energies for the *S*_1_, *S*_2_ and *T*_1_ states are comparable between the two active spaces (within 0.12 eV), while the *T*_2_ state exhibits significant energy differences between the active spaces for conformers C1 and C2 (∼0.71 eV). The *S*_1_ and *T*_1_ states correspond to the (*n*_O_, π*) excitation, while the *S*_2_ and *T*_2_ states correspond to the (*n*_S_, π*) excitation. However, the *T*_2_ state has non-negligible contributions from the lone pair orbitals of the peroxide oxygens. Although the *T*_2_ energy differences for C1 and C2 with the two active spaces are likely caused by these orbitals, it does not justify the use of the extended space because of its high computational cost. The main reason is that, as discussed in the next section, the triplet states do not play a significant role in the excited-state chemistry of the molecule after population of the *S*_1_ state. Therefore, the reduced active space was selected for the subsequent calculations.

**Table 2:** Vertical Excitation Energies (in eV) for the Lowest Lying Singlet (*S*_1_, *S*_2_, *S*_3_) and Triplet (*T*_1_, *T*_2_) Excited States of Conformers C1 to C4^a^

	extended space				reduced space			
state	C1	C2	C3	C4	C1	C2	C3	C4
*S*_1_	4.76	4.76	4.71	4.71	4.73	4.73	4.69	4.69
*S*_2_	5.65	5.65	5.84	5.84	5.77	5.77	5.89	5.89
*S*_3_	6.51	6.51	6.74	6.74	6.60	6.60	6.60	6.60
*T*_1_	4.50	4.50	4.44	4.44	4.51	4.51	4.45	4.45
*T*_2_	4.66	4.66	5.24	5.24	5.37	5.37	5.53	5.53

a Calculated with MS-CASPT2 (with IPEA = 0.25 a.u.) using the extended and reduced active spaces.

### Photochemistry: Static Approach

The static analysis of the photochemistry of HPMTF was performed on conformers C1 to C4. Table 2 shows that the *S*_1_ and *T*_1_ states were close in energy (∼0.2 eV), which might be relevant to the HPMTF photochemistry. Therefore, we optimized the *S*_1_ and *T*_1_ minima using TD-DFT ((U)­DFT for *T*_1_), SA-CASSCF and XMS-CASPT2. It is established that the dynamical correlation is relevant for excited state geometry optimizations, especially for surface crossing regions.^70^ Nevertheless, we have also used the SA-CASSCF method for the geometry optimizations to evaluate the effect of both static and dynamic correlation in the geometries. The reason is to verify whether static correlation would be enough or not for subsequent dynamics simulations. The molecular structures of the *S*_1_ minima at the XMS-CASPT2 level for conformers C1 to C4 are represented in Figure 7.

**Figure 7:**
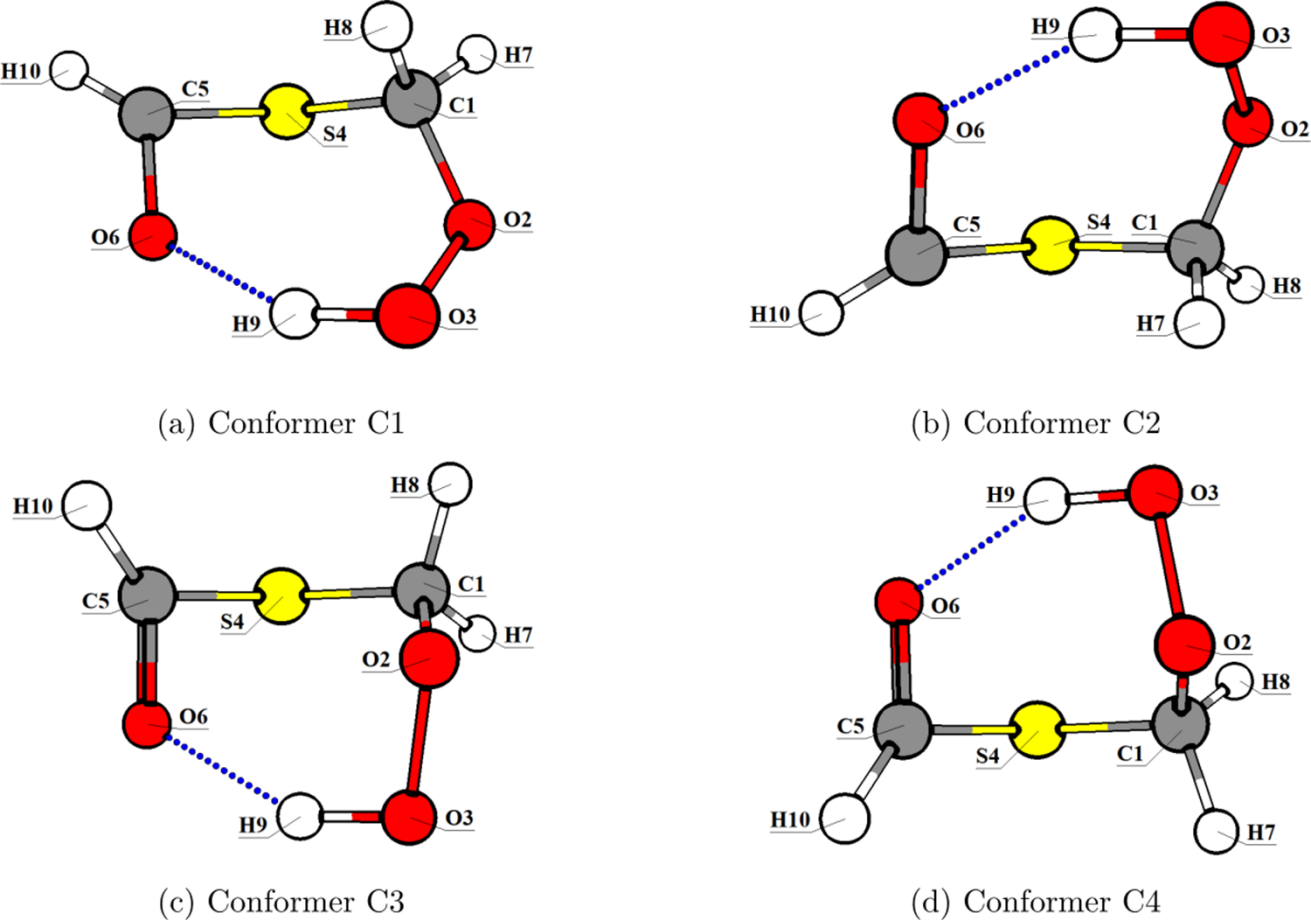
Molecular representations of the *S*_1_ minima at XMS-CASPT2­(10,8) level for conformers (a) C1, (b) C2, (c) C3, and (d) C4.

The pseudoenantiomeric relationships between conformers were preserved in the excited states. The optimized *S*_1_ structures shifted away from planarity to a pyramidal shape at the carbonyl group, which is a typical behavior in organic compounds with the given functional group. The C3 and C4 conformers formed H-bonds in the *S*_1_ minimum similar to those found in the minima of the C1 and C2 conformers.

For the C1 to C4 conformers, the TD-DFT optimizations of the *S*_1_ state converged to geometries analogous to those obtained with the XMS-CASPT2 method (RMSD ≈ 0.25–0.43 Å in Table S3). SA-CASSCF optimizations, however, converged to equivalent structures where the H9 atom did not form the hydrogen bond (RMSD ≈ 1.35–1.86 Å in Table S3). This indicates that the dynamical correlation introduced by CASPT2 is relevant to the geometry optimization in this case.

The optimized *T*_1_ structures of conformers C1 and C2 were equivalent to their *S*_1_ counterparts across all three methods, whereas C3 and C4 converged to new minima (Figure S7).

According to Khan et al.,^23^ HPMTF photolysis proceeds via dissociation of the O2–O3 or S4–C5 bonds. To initially explore the plausibility of such excited-state dissociations, we performed rigid scans along both bond coordinates, starting from the ground-state optimized geometry and using the C4 conformer as representative system.

The scan for the O2–O3 bond dissociation is represented in Figure 8. The states *S*_1_ and *T*_1_ in the FC region showed (*n*_O_, π*) character and bonding behavior. However, a dissociative triplet state could be observed crossing the rest of excited states, exhibiting (σ, σ*) character associated to the O2–O3 bond (black dots in Figure 8).

**Figure 8:**
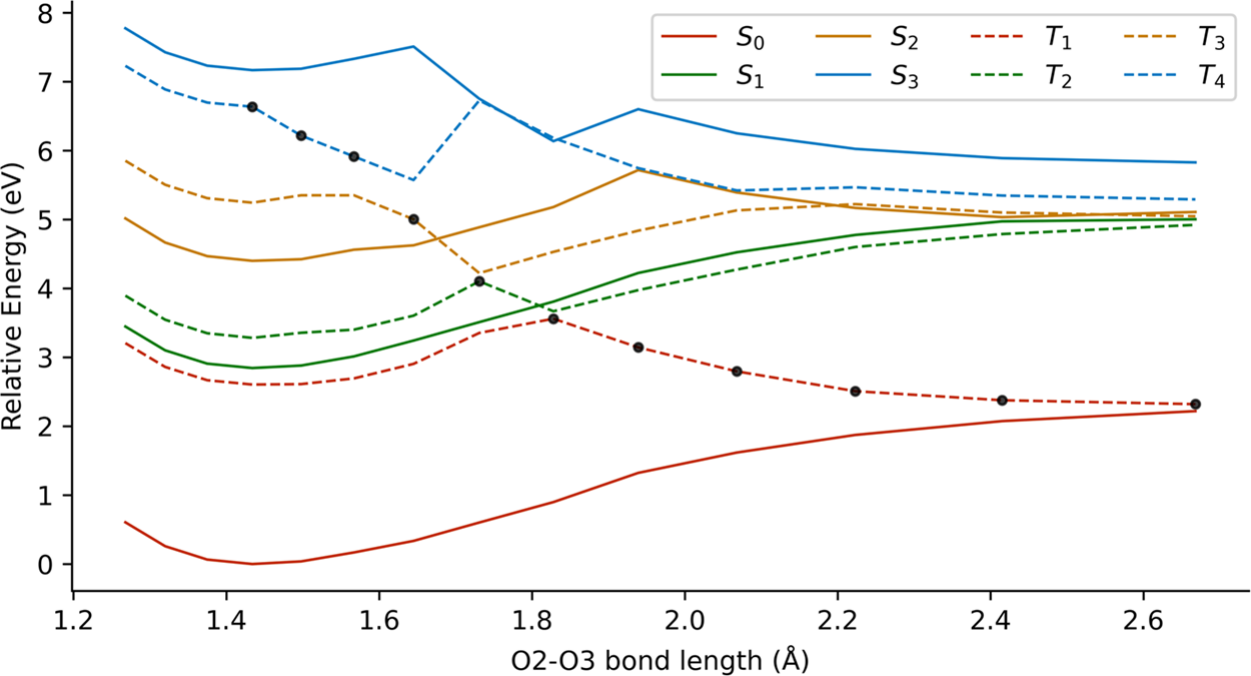
Rigid scan of the O2–O3 bond dissociation at the XMS-CASPT2­(10,8) level for conformer C4. The black dots represent the (σ, σ*) character.

Therefore, the dissociation of the O2–O3 bond should follow the *S*_1_ state until reaching a crossing region with *T*_2_ and undergoing intersystem crossing (ISC). Then, the molecule can access *T*_1_ through another crossing region, leading to the breaking of the O2–O3 bond. To estimate the ISC rate constant, we calculated the spin–orbit couplings between the states *S*_1_ and *T*_2_ at the geometry closest to the state crossing of the rigid scan. The absolute values were approximately 5 cm^–1^ (see Table S4 for exact values), which suggests that the ISC is a relatively slow process. We found this value to be consistent with our observations because the orbitals involved in each state have the same symmetry nature and therefore ISC is not favored according to the El-Sayed rules.^71^

Figure 9 shows the rigid scan of the S4–C5 bond dissociation, where the *S*_1_ state becomes dissociative after surpassing an energy barrier of 0.80 eV (located at ca. 2.3 Å). In this region, the *S*_0_ state approaches *S*_1_ (energy gap of 0.75 eV at ca. 3.0 Å), suggesting the presence of a CI. Regarding the triplet states, we observed that both *T*_1_ and *T*_2_ had analogous dissociative behavior with respect to *S*_1_ and the *S*_1_ and *T*_2_ states are degenerate.

**Figure 9:**
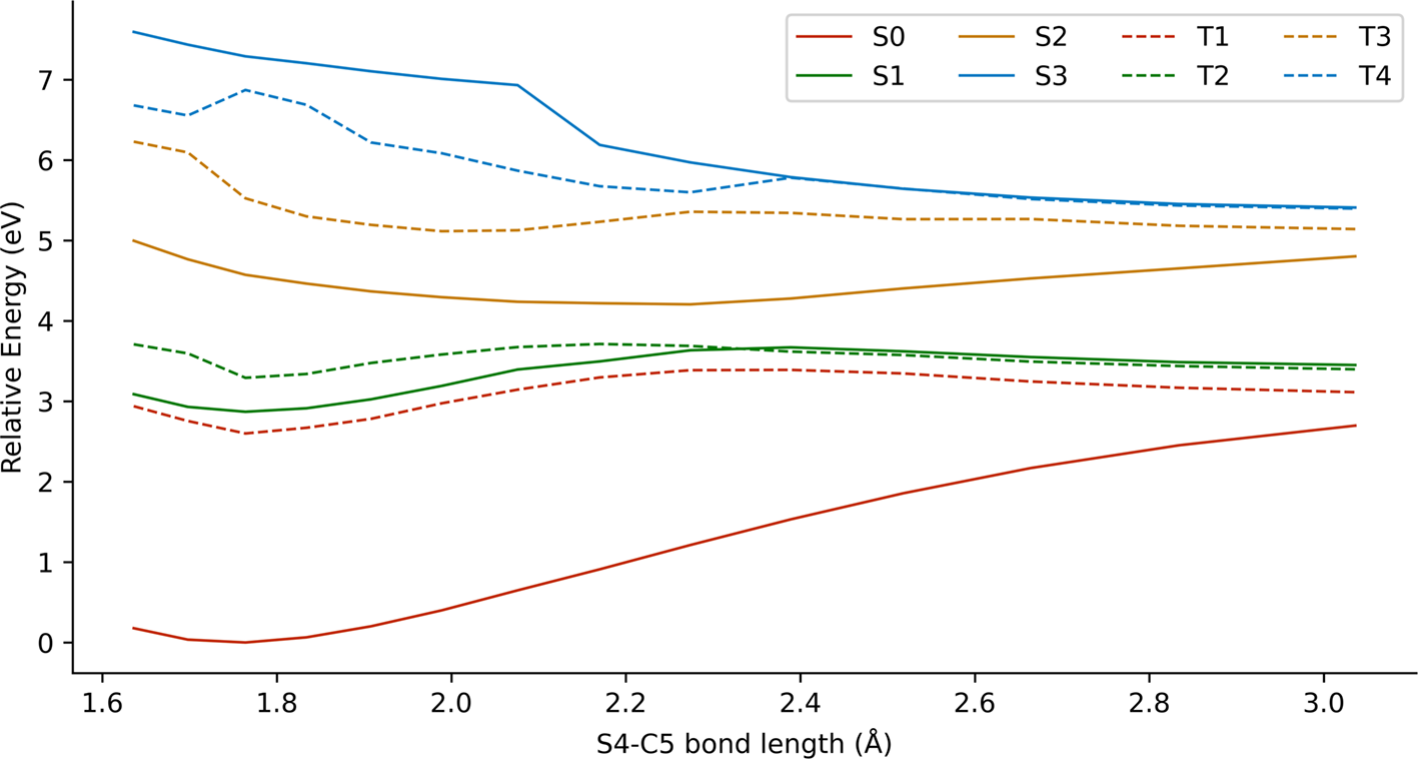
Rigid scan of the S4–C5 bond dissociation at the XMS-CASPT2­(10,8) level for conformer C4.

Following these initial scans, we optimized the geometries of the relevant critical points found in the analysis: the CI between *S*_0_ and *S*_1_ along the S4–C5 bond stretching, and the state crossing between *S*_1_ and *T*_2_ along the O2–O3 bond stretching. Each point was characterized using two approaches: XMS-CASPT2//SA-CASSCF and XMS-CASPT2//XMS-CASPT2.

For the *S*_1_/*T*_2_ crossing point, both optimization methods yielded similar structures (Figure 10). By comparing the XMS-CASPT2 optimized structure with the corresponding singlet–triplet crossing point in the rigid scan structure discussed above, we found that the former has even lower absolute values for the spin–orbit couplings, around 0.15 cm^–1^ (see Table S4 for exact values), indicating an even slower ISC rate constant.

**Figure 10:**
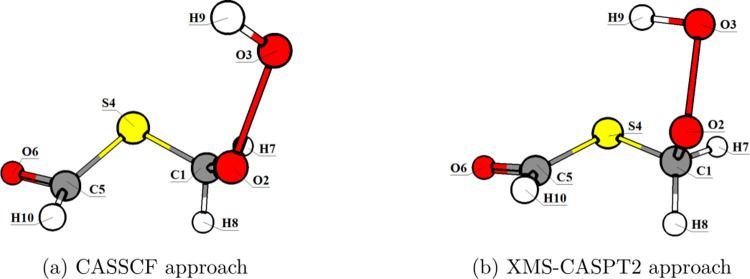
Molecular structures at the crossing point between *S*_1_ and *T*_2_ using the (a) SA-CASSCF­(10,8) and (b) XMS-CASPT2­(10,8) approaches.

In the case of the CI, the two approaches did not converge to similar geometries. SA-CASSCF obtained a structure resembling the *S*_1_ minimum shown in Figure 7, whereas XMS-CASPT2 led to a structure where S4–C5 dissociation happened (see Figure 11). The topologies of the CIs are classified as sloped (*P* = 13.545) and single-path (*B* = 3.000) for the SA-CASSCF optimized structure, and peaked (*P* = 0.359) and bifurcating (*B* = 0.783) for the XMS-CASPT2 optimized geometry. As seen in Figure 9, the potential energy curve (PEC) of *S*_0_ and *S*_1_ get closer at the dissociation limit, which is in agreement with the optimized CI.

**Figure 11:**
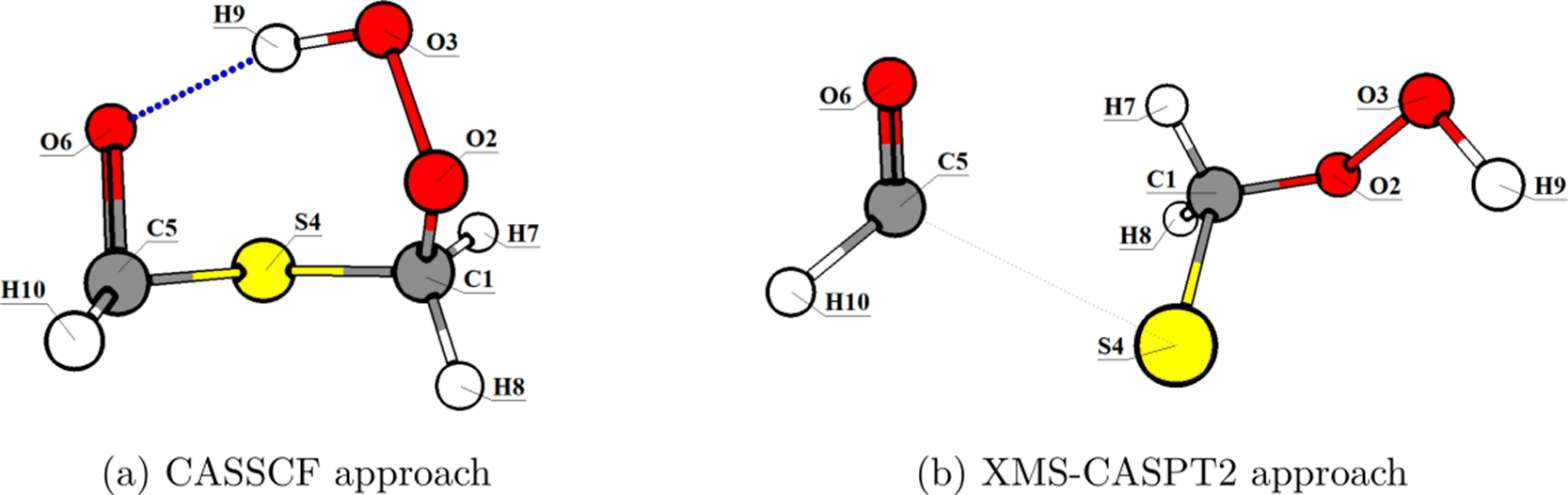
Molecular structures at the CI between *S*_0_ and *S*_1_ using the (a) SA-CASSCF­(10,8) and (b) XMS-CASPT2­(10,8) approaches.

The XMS-CASPT2 energy difference between *S*_0_ and *S*_1_ was equal to 0.2 eV for the SA-CASSCF optimized structure, which does not correspond to a CI (around 0.1 eV or less). Therefore, we determined that the consideration of dynamical correlation is also relevant to accurately describe the excited state chemistry of HPMTF.^72^

Finally, we performed geodesic-interpolated scans to model the potential energy surface of the excited states and select the theoretical method for the subsequent NAMD simulations. The interpolated paths connected the ground-state optimized structures of each conformer to the dissociated structure obtained for the CI at the XMS-CASPT2 level (see Figure S8).

The PECs for conformers C1 and C4 have negligible energy barriers for the S4–C5 bond dissociation, while conformers C2 and C3 have energy barriers of 0.92 and 0.78 eV, respectively. However, the latter barriers are not associated to the S4–C5 bond dissociation, but to a spatial rearrangement of the oxygen atoms of the peroxide group. This rearrangement is caused by the symmetric relationships of the conformers because the CI geometry was optimized from conformer C4. We expect that optimizing the CI from conformers C2 or C3 would lead to PECs where the behavior observed here would be reversed. Nevertheless, they were not calculated because the photochemistry of conformers C1 and C2, or C3 and C4, is identical to each other, as discussed in previous sections.

Focusing on conformers C1 and C4, we generated two additional paths for these conformers, and we calculated the PECs using several methods to compare their performance: XMS-CASPT2, CC2 and TD-DFT/ωB97-XD. These new paths connected three relevant points for each conformer: *S*_0_ minimum, *S*_1_ minimum, and the CI obtained from conformer C4. As discussed above, we omitted the calculation of triplet states in these investigations due to their secondary role.

First, conformer C1 (Figure 12) exhibited an energy barrier for the S4–C5 bond dissociation of 0.28 eV at the XMS-CASPT2 level. CC2 and TD-DFT/ωB97-XD overestimated the energy barrier, 0.59 and 0.75 eV, respectively. CC2 described more accurately the PEC than TD-DFT/ωB97-XD, but the energy gap at the CI for TD-DFT/ωB97-XD (0.23 eV) was smaller than for CC2 (0.37 eV). Considering the significantly higher computational cost of CC2 over TD-DFT/ωB97-XD, we selected TD-DFT/ωB97-XD for the NAMD simulations.

**Figure 12:**
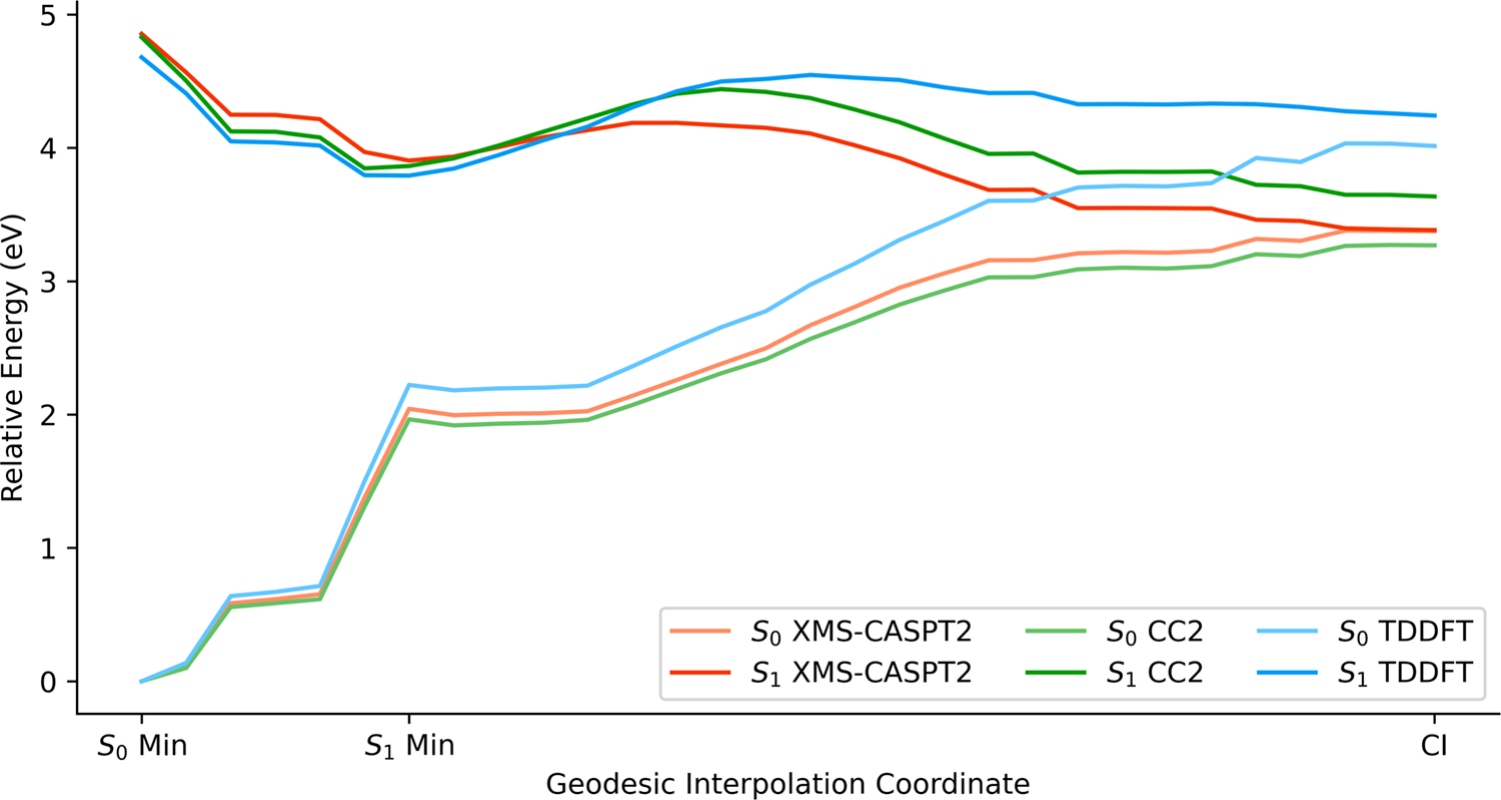
PECs for the S4–C5 bond dissociation of conformer C1 using XMS-CASPT2, CC2, and TD-DFT/ωB97-XD. The path was generated by interpolating two sets of points: the ground-state minimum (*S*_0_ Min) and the first singlet excited state minimum (*S*_1_ Min), as well as *S*_1_ Min and the CI between *S*_0_ and *S*_1_. The points used across all three methods were obtained with DFT/ωB97-XD (*S*_0_ Min) and XMS-CASPT2­(10,8) (*S*_1_ Min and CI).

Second, conformer C4 (Figure 13) also exhibited an energy barrier for the S4–C5 bond dissociation. However, the barrier was significantly higher, at 2.41 eV for XMS-CASPT2 and 2.26 eV for TD-DFT/ωB97-XD. Additionally, we generated a second path for conformer C4 where we substituted the *S*_1_ minimum structure with the *T*_1_ minimum structure (see Figure S9). This path had lower energy barriers with 0.61 eV for XMS-CASPT2 and 1.37 eV for TD-DFT/ωB97-XD. The main difference between the two pathways is the H-bond that is formed in the *S*_1_ minimum. This H-bond is likely the cause of the increased energy barrier.

**Figure 13:**
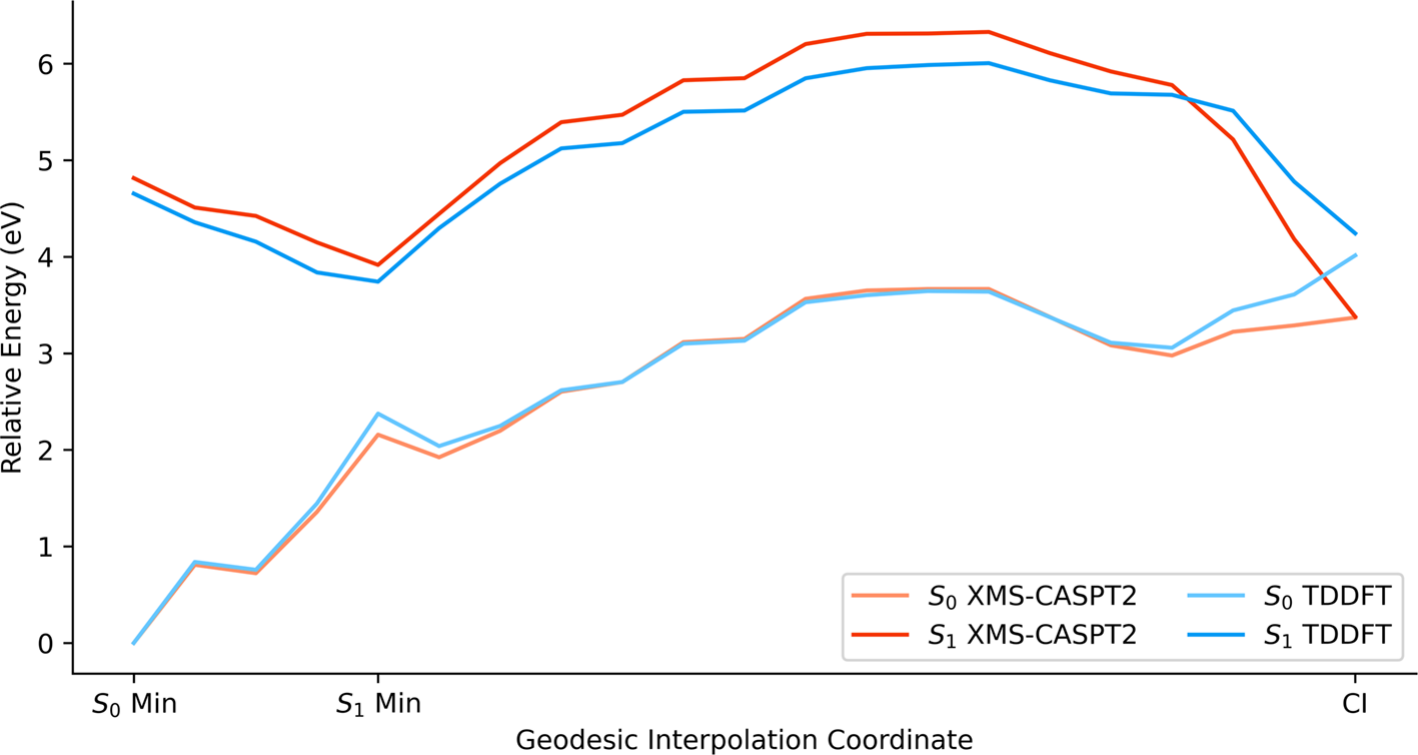
PECs for the S4–C5 bond dissociation of conformer C4 using XMS-CASPT2 and TD-DFT/ωB97-XD. The path was generated by interpolating two sets of points: the ground-state minimum (*S*_0_ Min) and the first singlet excited state minimum (*S*_1_ Min), as well as *S*_1_ Min and the CI between *S*_0_ and *S*_1_.

### Photochemistry: Dynamic Approach

The dynamic calculations of the system included conformers C1 to C4. Although the number of initial conditions calculated was equal for each conformer, the number of trajectories varied. The trajectories simulated for each conformer were 25, 26, 18 and 10, respectively.

We began by analyzing the complete ensemble of trajectories before examining specific dynamical features with smaller sets of simulations. One of the most relevant properties that can be extracted from the NAMD simulations is the lifetime of the excited state. The excited state decay rate usually follows an exponential function or a multiexponential function depending on the number of relaxation mechanisms. Here, we fit the *S*_1_ state population over time to the multiexponential function given by eq 3:

$$y=\sum_{i=1}^{n}(\alpha_{i}e^{-\lambda_{i}t})+\beta$$ 3

where *y* is the *S*_1_ state population, *n* is the number of exponential functions, *t* is the time (in fs), α_*i*_ and λ_*i*_ are the coefficient and decay rate constant of the exponential functions, and β is the remaining population of the excited state. The decay rate constant, λ, of an exponential decay function can be used to calculate its lifetime using eq 4:

$$\tau =\frac{1}{\lambda }$$ 4

where τ is the lifetime, in fs. Thus, combining eqs 3 and 4, we can express the exponential decay in lifetime terms as given in eq 5:

$$y=\sum_{i=1}^{n}(\alpha_{i}e^{-t/\tau_{i}})+\beta$$ 5

We performed the fitting of the *S*_1_ population decay using up to *n* = 3 exponential functions. The biexponential function, given in eq 6, obtained the best fitting (*R* ^2^ = 0.9917) among all three functions.

$$y=0.596e^{-t/79.76}+0.442e^{-t/470.57}-0.044$$ 6

Figure 14 represents the population of the electronic states included in the NAMD simulations over time and the biexponential curve fitted to the decay of the *S*_1_ state.

**Figure 14:**
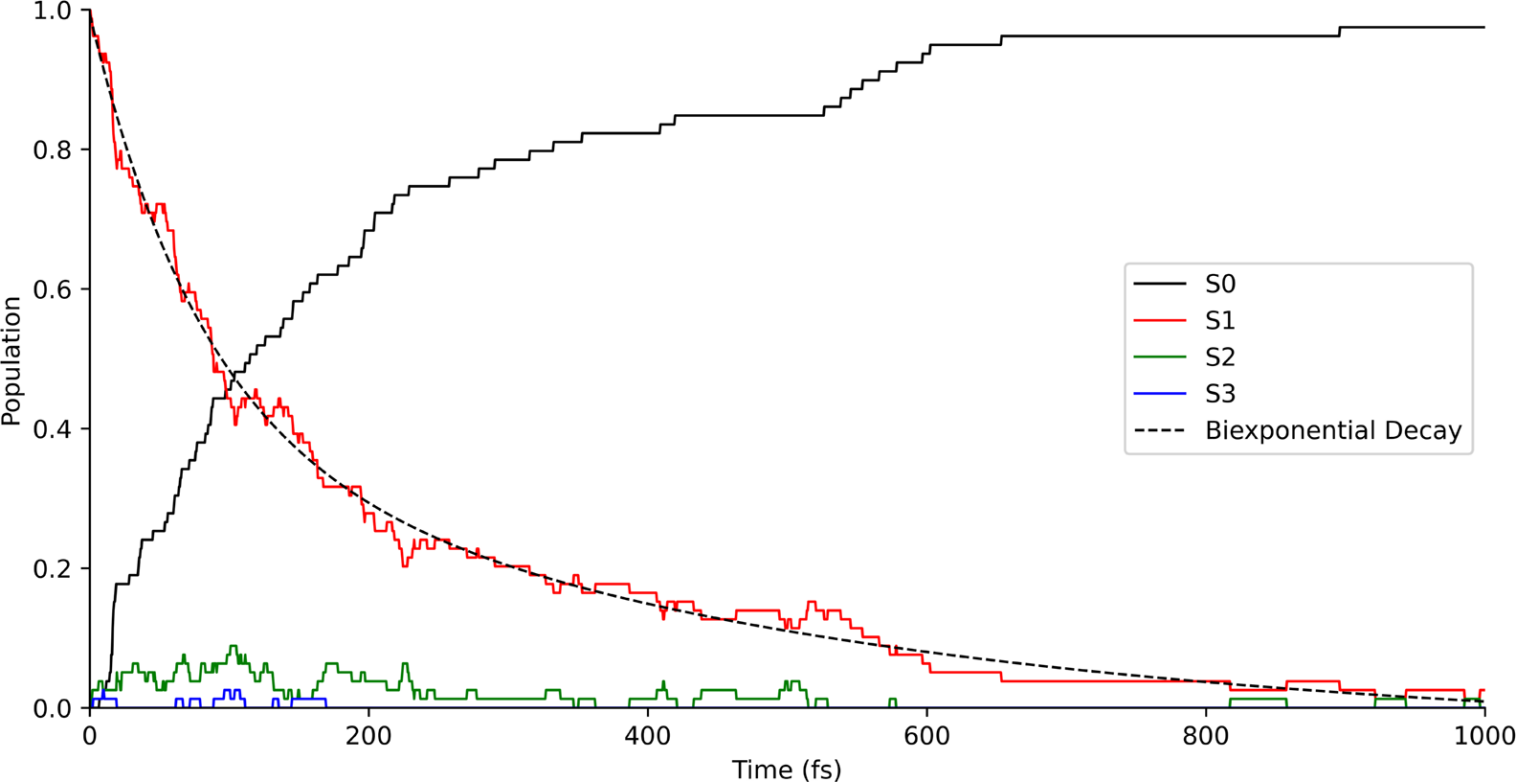
Population of states over time (in fs) for the four singlet states included in the NAMD simulations. The dashed line represents the biexponential function fitted to the *S*_1_ decay.

From this analysis, we observe two different mechanisms: a faster process with an approximated lifetime of 79.76 fs, and a slower process with an estimated lifetime of 470.57 fs. Both mechanisms are considered to be relatively fast relaxation processes, which further proves that the slow ISC process should not play an important role in the photochemistry of HPMTF.

Now, we examined the photochemical pathways of HPMTF. As expected from our static study, the most relevant pathway was the S4–C5 dissociation. 37 trajectories (∼47% of the total) led to S4–C5 dissociation. The population decay corresponding to the S4–C5 dissociation was fitted to a monoexponential function with an estimated lifetime of 150.32 fs (*R* ^2^ = 0.9730). Moreover, additional minor dissociation pathways were also found: approximately 7% of trajectories (6 trajectories) led to C1–S4 cleavage, and 14% (11 trajectories) led to O2–O3 cleavage. The O2–O3 dissociation was not taken into account in the static approach because the *S*_1_ state was characterized as a bonding state in our rigid scan (see Figure 8). Therefore, other collective coordinates besides the S4–C5 bond are also involved in driving the system toward this dissociation. Taking all three pathways into account, we obtained a dissociation quantum yield of ϕ = 0.67.

Next, we discuss the trajectories that did not undergo bond breaking during the simulation. We observed a nonreactive pathway through the transfer of a hydrogen atom between the O9 and O6 atoms, herein referred to as H-transfer photostabilization, in ∼14% (11 trajectories). Although we cannot estimate the lifetime of this process due to insufficient statistical significance, it is worth noting that the process was substantially faster than the other pathways, with the slowest trajectory lasting around 87 fs. The remaining ∼18% (14 trajectories) relaxed to the ground state through distorted structures. For example, elongation of the C1–S4, S4–C5, and O3–H9 bond distances.

Taken all together, the S4–C5 bond dissociation and H-transfer photostabilization account for 61% of trajectories, agreeing well with the coefficient of the fast biexponential decay component (0.596). Thus, this suggests that the fast exponential decay is primarily associated with S4–C5 bond cleavage and H-transfer photostabilization. On the other hand, the slower component is likely related to the other processes taking place at the excited state.

While the dynamics analysis did not differentiate among the four conformers, the conformer pairs exhibit one key distinction: the H-bond. Only the isomers C1 and C2 present a H-bond between H9 and O6. According to the findings, C3 and C4 conformers have a higher ratio of S4–C5 bond dissociation (ϕ = 0.64) compared to C1 and C2 (ϕ = 0.37), which is likely due to the absence of an H-bond.

### Photolysis Rates and Global Impact

Finally, we discuss the effects on the atmospheric sulfur cycle according to our results. Considering the initial absorption energies of the nonadiabatic molecular dynamics, we establish that our quantum yield value is valid for the range of wavelengths between 245 and 300 nm, approximately, which includes the atmospheric window of interest in this work. Thus, we set a lower limit for the dissociative quantum yield of ϕ = 0.67 and an upper limit of ϕ = 1, and estimate the photolysis rates using eq 1. The calculated photolysis rate constants and associated lifetimes are given in Table 3. It is relevant to note that the photolysis rate was calculated under maximum irradiation conditions (see Computational Details section).

**Table 3:** Photolysis Rates and Lifetimes for HPMTF

quantum yield, ϕ (λ)	photolysis rate, *J* (s^–1^)	lifetime, τ (h)
ϕ(245–300 nm) = 1	1.41 × 10^–5^	19.76
ϕ(245–300 nm) = 0.67	9.42 × 10^–6^	29.49

The lifetime of ∼20–30 h proves that photolysis is a minor loss term for HPMTF compared to other sinks such as cloud uptake (τ ≈ 1–2 h),^22,25^ and OH oxidation (τ ≈ 14 h).^16,20^ In conditions of high humidity, the loss of HPMTF is large, and the photolysis contribution is thus, negligible. In low humidity conditions, HPMTF plays a role as sulfur reservoir with a lifetime over 0.5 days and the photolysis contributes as a minor loss source.

## Conclusions

In this work, we discuss the photochemical relevance of the interaction between light and HPMTF in the marine atmosphere. The gas-phase absorption spectrum was calculated and compared to previous values which overestimated the light absorption of HPMTF.^23,27^ Static and dynamic studies of the PESs of HPMTF were performed to understand its photochemistry. The results showed multiple dissociation pathways; however, the most significant was the thioformate bond dissociation (S4–C5). The quantum yield of the photolytic processes (ϕ = 0.67), the photolysis rate (*J* = 9.42 × 10^–6^ s^–1^) and its photolytic lifetime (τ ≈ 30 h) were estimated. According to these results and the literature, we expect the photolysis of HPMTF to be a minor loss pathway in the marine atmosphere in dry (cloud free) conditions and negligible in humid conditions.

## Supplementary Material



## Data Availability

HPMTF dynamics (TAR.GZ file containing nonadiabatic molecular dynamics data) is available at 10.5281/zenodo.17236561.

## Notes

Supporting Information: Cartesian RMSD values for relevant molecular structures (Tables S1 and S3), *S*_1_ absorption energies for conformers C1 to C10 (Table S2), spin–orbit coupling values for *S*_1_ *–T*_2_ crossing geometries (Table S4), natural orbitals considered in the complete active space (CAS) of the multiconfigurational calculations (Figures S1 and S2), molecular structures of the HPMTF ground-state conformers (Figures S3 and S4), gas-phase absorption spectra of the different conformers (Figure S5), gas-phase absorption spectra of HPMTF in the 100–300 nm energy range (Figure S6), molecular structures of HPMTF conformers at the *T*_1_ minimum (Figure S7), and additional potential energy curves along the S4–C5 bond breaking coordinate (Figures S8 and S9) (PDF)
