# Supplementary material for: Excited-State Chemistry of Hydroperoxymethyl Thioformate in the Troposphere
Source: J Phys Chem A. 2026 Jan 26;130(5):1046–59. doi: 10.1021/acs.jpca.5c07092 (PMC12884519; doi:10.1021/acs.jpca.5c07092)
Supplement: Supplementary file 1 [file jp5c07092_si_001.pdf]

Supporting Information: Excited-State Chemistry of Hydroperoxymethyl Thioformate in the Troposphere

David Catalán-Fenollosa,[†] Mariana Telles do Casal,[‡] Javier Carmona-García,[¶] Alfonso Saiz-Lopez,[§] Daniel Escudero,[*,‡] and Daniel Roca-Sanjuán[*,†]

†*Institut de Ciencia Molecular, Universitat de Valencia, 46071 Valencia, Spain*

‡*Quantum Chemistry and Physical Chemistry Division, Department of Chemistry, KU Leuven, Celestijnenlaan 200F, 3001 Leuven, Belgium*

¶*Centre for Computational Chemistry, School of Chemistry, University of Bristol, Bristol BS8 1TS, U.K.*

§*Department of Atmospheric Chemistry and Climate, Institute of Physical Chemistry Blas Cabrera, CSIC, 28006 Madrid, Spain*

E-mail: daniel.escudero@kuleuven.be; daniel.roca@uv.es

Table S1: Cartesian RMSD values (Å) for ground-state optimizations for each method with respect to DF-CCSD(T)-F12b/jun-cc-pVDZ for conformer C3.

M06-2X	B3LYP	PBE0	CAM-B3LYP	ωB97-XD	MP2	CCSD
0.2872	0.2771	0.2724	0.2185	0.2092	0.2115	0.1936

Table S2: S_1 vertical excitation energies of exchange correlation functionals and CC2 for conformers C1 to C10.

	C1	C2	C3	C4	C5	C6	C7	C8	C9	C10
CAM-B3LYP	4.63	4.63	4.61	4.61	4.62	4.63	4.58	4.58	4.53	4.53
M06-2X	4.60	4.60	4.59	4.57	4.57	4.57	4.53	4.54	4.49	4.49
PBE0	4.62	4.62	4.64	4.64	4.65	4.65	4.60	4.60	4.56	4.56
ωB97-XD	4.63	4.63	4.62	4.62	4.63	4.63	4.58	4.58	4.54	4.54
CC2	4.82	4.82	4.82	4.82	4.83	4.83	4.80	4.80	4.76	4.76

Table S3: Cartesian RMSD values (Å) for excited-state optimizations using different methods with respect to XMS-CASPT2(10,8) for conformers C1 to C4 and excited states S_1 and T_1.

Conformer	State	TD-DFT	CASSCF(10,8)	XMS-CASPT2(10,8)
C1	S_1	0.4231	1.3536	0.0000
	T_1	0.2695	1.2985	0.3099
C2	S_1	0.4312	1.3499	0.0000
	T_1	0.2775	1.2953	0.3240
C3	S_1	0.2576	1.8576	0.0000
	T_1	3.5350	3.6199	3.3518
C4	S_1	0.2529	1.8584	0.0000
	T_1	3.5363	3.6153	3.3590

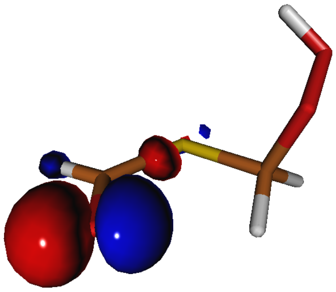

(a) n(O6)

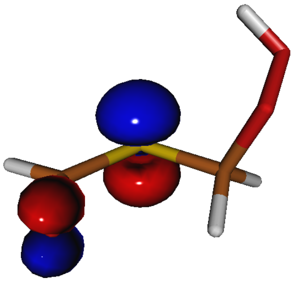

(b) n(S4)

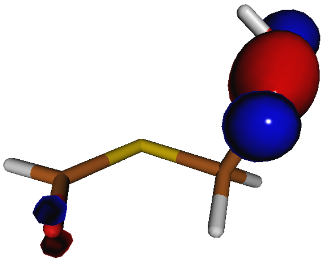

(c) σ(O2-O3)

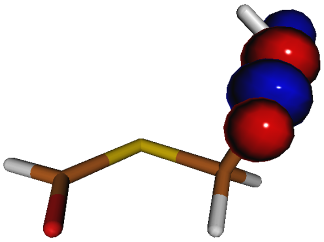

(d) σ^*(O2-O3)

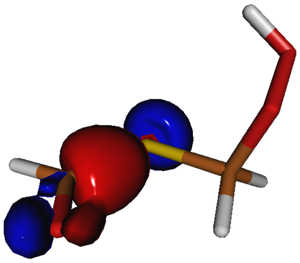

(e) σ(S4-C5)

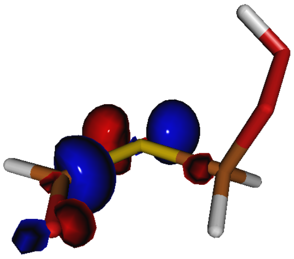

(f) σ^*(S4-C5)

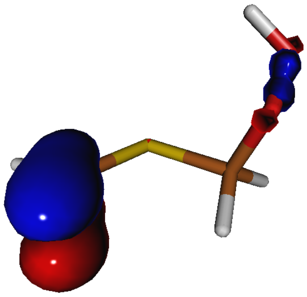

(g) π(C5-O6)

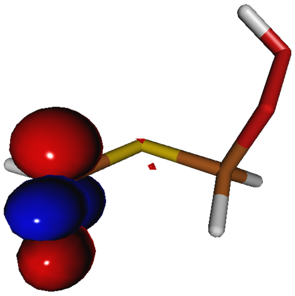

(h) π^*(C5-O6)

Figure S1: Representation of the molecular orbitals included in both CAS(16,12) and CAS(10,8) active spaces.

Table S4: Spin-orbit coupling absolute values, in cm^{-1}, for the rigid scan structure and the optimized S_1-T_2 crossing structure.

	S_1-$T_2(M_s = -1)$	S_1-$T_2(M_s = 0)$	S_1-$T_2(M_s = +1)$
Rigid Scan	5.492	3.663	5.492
Optimized Crossing	0.150	0.117	0.150

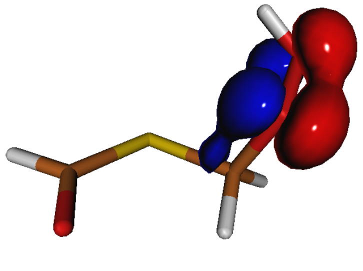

(a) n(O2)/n(O3)

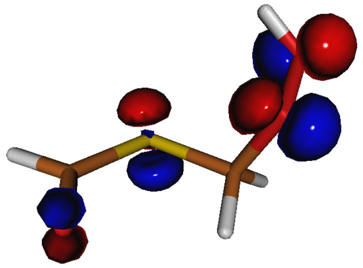

(b) n(O2)/n(O3)

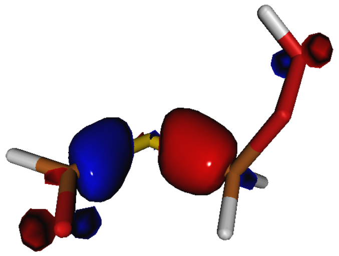

(c) σ(C1-S4)

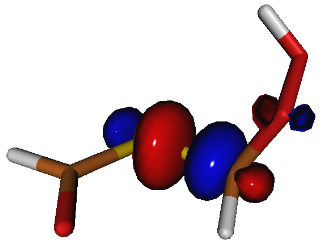

(d) σ^*(C1-S4)

Figure S2: Representation of the molecular orbitals included exclusively in the CAS(16,12) active space, and not in the CAS(10,8) active space.

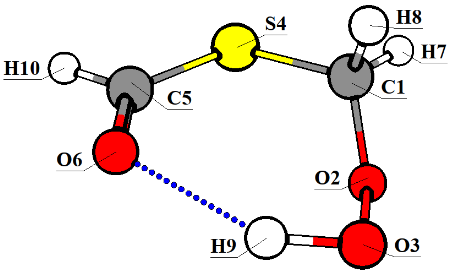

(a) Conformer C1

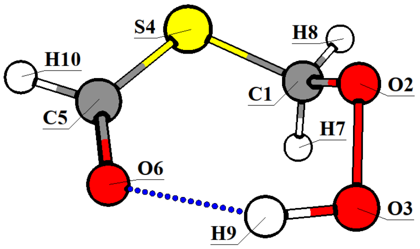

(b) Conformer C2

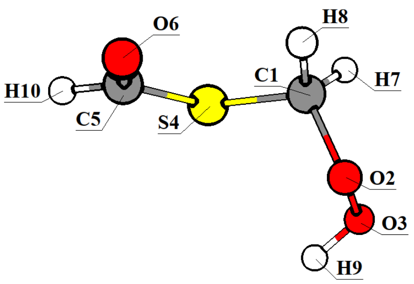

(c) Conformer C3

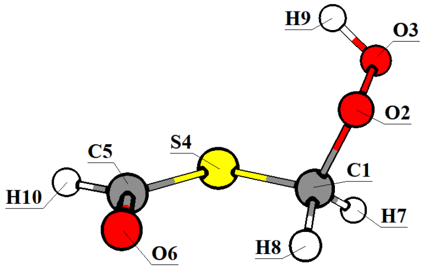

(d) Conformer C4

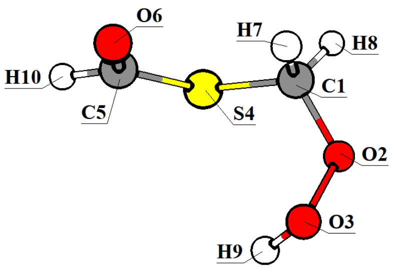

(e) Conformer C5

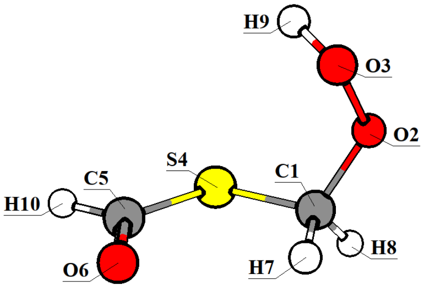

(f) Conformer C6

Figure S3: Molecular representations of ground-state optimized structures of conformers C1 to C6 at DFT/ωB97-XD with atom numbering and labelling.

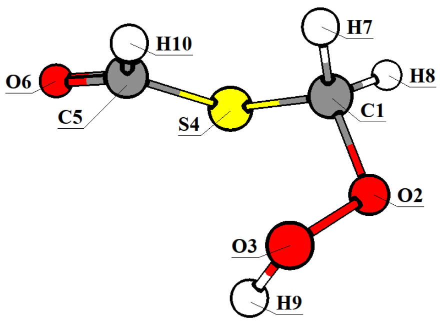

(a) Conformer C7

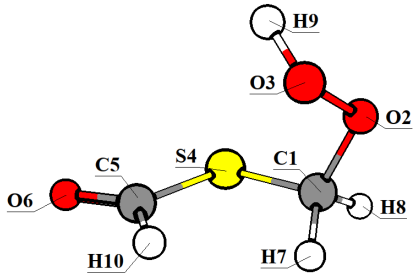

(b) Conformer C8

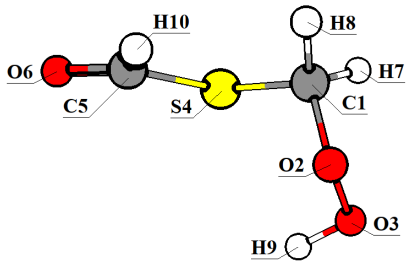

(c) Conformer C9

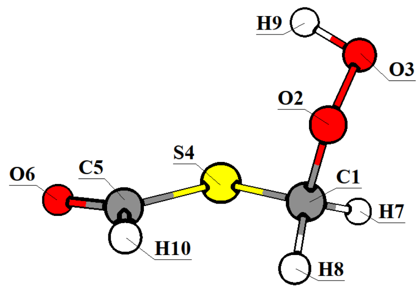

(d) Conformer C10

Figure S4: Molecular representations of ground-state optimized structures of conformers C7 to C10 at DFT/ωB97-XD with atom numbering and labelling.

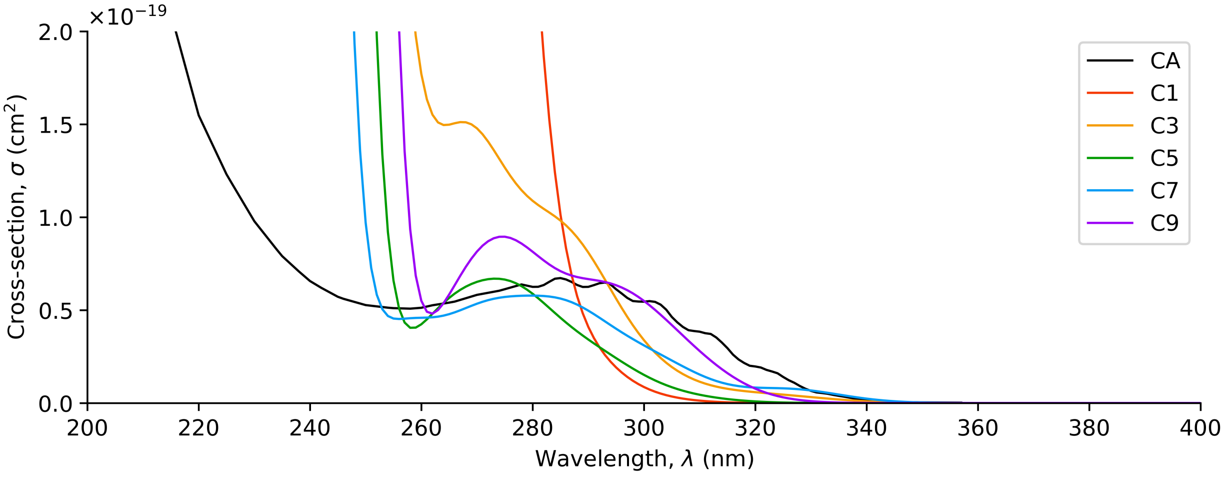

Figure S5: Gas-phase UV-Vis absorption spectra of the HPMTF conformers C1, C3, C5, C7 and C9 and chromophore approximation (CA). The spectra are calculated using MS-CASPT2(16,12) with IPEA correction.

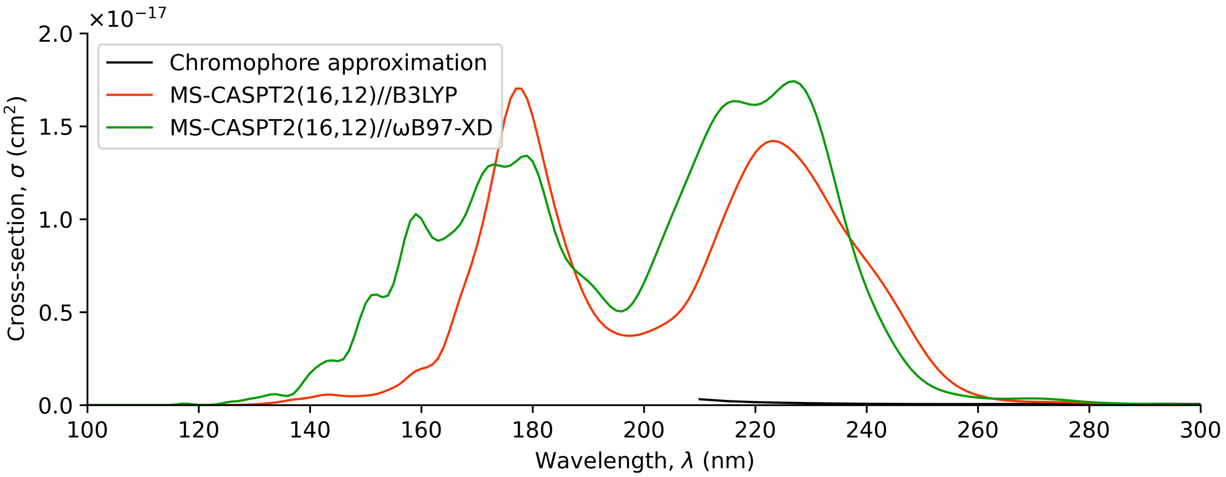

Figure S6: Gas-phase UV absorption spectra of HPMTF. The black curve corresponds to the chromophore approximation. The red and green curves correspond to calculated spectra with NEA using B3LYP and ωB97-XD, respectively. The spectra are calculated using MS-CASPT2(16,12) with IPEA correction.

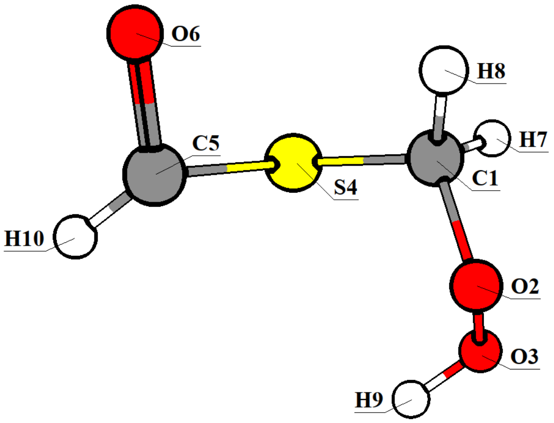

(a) Conformer C3

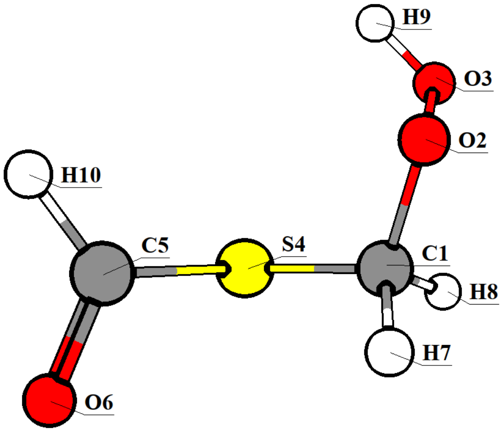

(b) Conformer C4

Figure S7: Molecular representations of the T_1 minima at XMS-CASPT2(10,8) level.

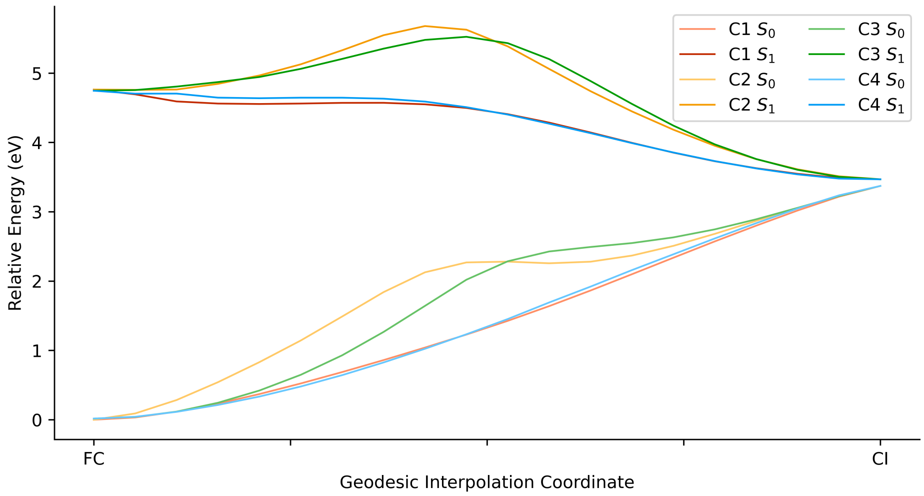

Figure S8: Geodesic-interpolated scan at XMS-CASPT2(10,8) level for conformers C1 to C4. The initial point is the ground state minima of the conformers (FC) and the final point is the CI between S_0 and S_1.

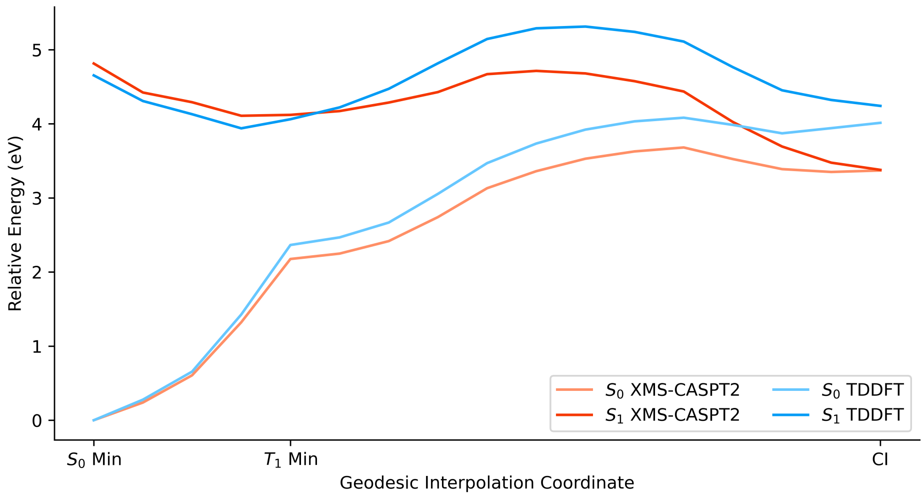

Figure S9: Potential energy curves for the S4-C5 bond dissociation of conformer C4 using XMS-CASPT2(10,8) and TD-DFT/ωB97-XD. The path was generated by interpolating two sets of points: the ground-state minimum (S_0 Min) and the first triplet excited state minimum (T_1 Min), as well as T_1 Min and the CI between S_0 and S_1.
